# Clusterin inhibition using OGX-011 synergistically enhances zoledronic acid activity in osteosarcoma

**DOI:** 10.18632/oncotarget.2308

**Published:** 2014-08-04

**Authors:** Francois Lamoureux, Marc Baud'huin, Benjamin Ory, Romain Guiho, Amina Zoubeidi, Martin Gleave, Dominique Heymann, Françoise Rédini

**Affiliations:** ^1^ Université de Nantes, Nantes atlantique universités, Laboratoire de Physiopathologie de la Résorption Osseuse et Thérapie des Tumeurs Osseuses Primitives, Nantes F-44035, France; ^2^ INSERM, UMR 957, Nantes F-44035, France; ^3^ LUNAM Université; ^4^ CHU de Nantes, Nantes F-44035, France; ^5^ Equipe labellisée LIGUE 2012, Nantes, cedex; ^6^ The Vancouver Prostate Centre, University of British Columbia, Vancouver, BC, Canada

**Keywords:** zoledronic acid, clusterin, osteosarcoma, bone tumor

## Abstract

**Purpose:**

Despite recent improvements in therapeutic management of osteosarcoma, ongoing challenges in improving the response to chemotherapy warrants new strategies still needed to improve overall patient survival. Among new therapeutic approaches, zoledronic acid (ZOL) represents a promising adjuvant molecule to chemotherapy to limit the osteolytic component of bone tumors. However, ZOL triggers the elevation of heat shock proteins (Hsp), including Hsp27 and clusterin (CLU), which could enhance tumor cell survival and treatment resistance. We hypothesized that targeting CLU using siRNA or the antisense drug, OGX-011, will suppress treatment-induced CLU induction and enhance ZOL-induced cell death in osteosarcoma (OS) cells.

**Methods:**

The combined effects of OGX-011 and ZOL were investigated *in vitro* on cell growth, viability, apoptosis and cell cycle repartition of ZOL-sensitive or -resistant human OS cell lines (SaOS2, U2OS, MG63 and MNNG/HOS).

**Results:**

In OS cell lines, ZOL increased levels of HSPs, especially CLU, in a dose- and time-dependent manner by mechanism including increased HSF1 transcription activity. The OS resistant cells to ZOL exhibited higher CLU expression level than the sensitive cells. Moreover, CLU overexpression protects OS sensitive cells to ZOL-induced cell death by modulating the MDR1 and farnesyl diphosphate synthase expression. OGX-011 suppressed treatment-induced increases in CLU and synergistically enhanced the activity of ZOL on cell growth and apoptosis. These biologic events were accompanied by decreased expression of HSPs, MDR1 and HSF1 transcriptional activity. *In vivo*, OGX-011, administered 3 times a week (IP, 20mg/kg), potentiated the effect of ZOL (s.c; 50μg/kg), significantly inhibiting tumor growth by 50% and prolonging survival in MNNG/HOS xenograft model compared to ZOL alone.

**Conclusion:**

These results indicate that ZOL-mediated induction of CLU can be attenuated by OGX-011, with synergistic effects on delaying progression of osteosarcoma.

## INTRODUCTION

Osteosarcoma (OS) is the most common primary malignant bone tumor in both children and young adults with a peak of incidence at 18 years [1, 2]. The tumor generally develops on the average part of the long bones (femur and tibia), at the vicinity of an articulation, mainly the knee or the shoulder. Current therapeutic protocols consist in neoadjuvant poly-chemotherapy associated with conservative surgery. The long-term survival rate is 60% to 75% at 5 years for patients with localized tumor but drastically goes down to 25% if pulmonary metastases are detected at diagnosis. However, patients who do not respond to these conventional therapies have a poor prognosis. To significantly improve survival in children with OS, new therapeutic strategies targeting the molecular basis of OS and treatment resistance are required.

Zoledronic acid (ZOL), the second generation of nitrogen-containing bisphosphonates (N-BPs), represents a promising alternative to treat OS. N-BPs are stable synthetic analogues of endogenous pyrophosphate (PPi) [3]. N-BPs induces osteoclast apoptosis by inhibiting enzymes of the mevalonate pathway, especially farnesyl diphosphate synthase (FDPs) [4, 5]. ZOL is one of the most potent inhibitors of bone resorption in clinical use to treat osteoporosis and other osteoclast-mediated bone diseases such as primary bone tumors. Moreover, several studies using bisphosphonates especially N-BPs, have demonstrated promising results by selectively targeting OS tumor cells [6-8]. We have previously shown that ZOL is able to delay tumor progression, to prevent tumor relapse compared with chemotherapy alone and to prevent osteolytic lesions in preclinical models of OS [9]. Our results have provided the rationale supporting the French randomized phase III clinical protocol OS2006 (Clinicaltrials.gov NCT00470223), which combines ZOL with conventional therapy for adult and pediatric patients. Since then, other fundamental or preclinical studies have confirmed the beneficial effect of BPs in OS [6, 10, 11]. Indeed, ZOL directly affects the proliferation and survival of OS tumor cells *in vitro* [6, 12-14], making of ZOL an attractive therapy for the treatment of OS, targeting both tumor cells and bone microenvironment.

However, ZOL as a potential clinical application suggests an extensive and prolonged contact of cancer cells with this N-BP, which is localized and stored in tumor-bone microenvironment [15]. This continued exposure could increase the risk of resistance development [16]. Indeed, despite the widely use of bisphosphonates in the clinical management of cancer, few studies have reported ZOL-resistance development in cancer cells and that deserve to be explored. Development of treatment resistance is a common feature of most malignancies and the underlying basis for most cancer deaths. Treatment resistance evolves, at least, from selective pressures of treatment that collectively increase the apoptotic rheostat of cancer cells. Usually, the molecular mechanisms underlying resistance include overexpression of efflux pumps, inhibition of apoptosis (overexpression of anti-apoptotic members of the bcl-2 protein family), increased DNA damage repair, and alteration of drug targets [17] and cytoprotective chaperone networks [18, 19]. Indeed, several cytoprotective chaperones such as Heat Shock Protein-27 (Hsp27) or clusterin (CLU) are reported to play a protective function in tumor cells under stress condition such as conventional or targeted therapies [20, 21].

CLU is a heterodimeric stress-induced cytoprotective chaperone that inhibits protein aggregation in a manner analogous to small HSPs, and its promoter contains a 14-bp element recognized by the transcription factor HSF1 [22]. CLU is ubiquitously expressed but at variable levels depending on many severe physiological disturbances, including tumor formation. In human OS, CLU levels are overexpressed in OS to a variable extent, especially after conventional therapy and could be a valuable marker of aggressive extraosseous osteosarcoma [23]. Experimental and clinical studies associate CLU with development of treatment resistance, where CLU suppresses treatment-induced cell death in response to conventional chemotherapy, targeted therapies or radiation [19, 21, 24-27]. Overexpression of CLU in OS indicates drug resistance to conventional therapies [23, 27] and over-expression of CLU in prostate cancer cells accelerates progression after hormone- or chemo-therapy [19, 24], identifying CLU as an anti-apoptotic gene up-regulated by treatment stress that confers therapeutic resistance. OGX-011 is a second-generation phosphorothioate antisense oligonucleotide currently in late stage clinical development that potently inhibits CLU expression and enhances the efficacy of anticancer therapies in various human cancers [28, 29]. While targeting CLU synergistically enhances the cytotoxic effects of chemotherapy, a role for CLU has not been characterized in the context of ZOL treatment and resistance.

In the present study, we set out the hypothesis that ZOL induces a heat shock response with increased HSF1 activity and subsequently CLU expression, which functions as inhibitor of treatment-induced apoptosis, enhancing emergence of treatment resistance. Based on these data, knockdown of CLU using OGX-011 could potentiate the effect of zoledronic acid in osteosarcoma treatment.

## RESULTS

### Zoledronic acid induces expression of clusterin in osteosarcoma cells *in vitro* and *in vivo*

**Figure 1:**
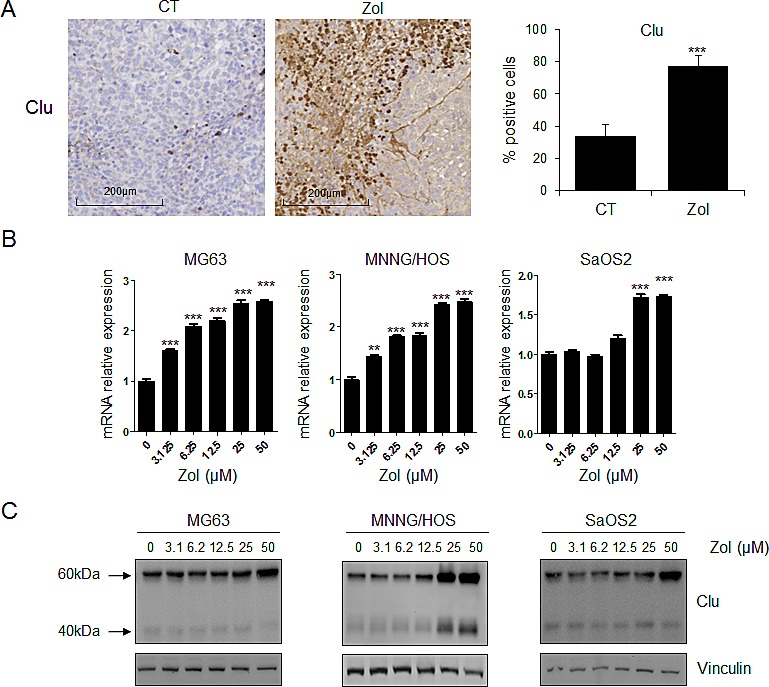
ZOL induces CLU expression in osteosarcoma *in vitro* and *in vivo* *A*, HOS-MNNG tumor cells were injected in paratibial site. Once tumors were palpable, mice were randomly assigned to vehicle (PBS) or ZOL (50ug/kg, s.c.) three times per week for 3 weeks. Tumors were collected after 3 weeks and CLU expression was evaluated by immunohistochemical analysis. Specimens were scored and estimated in % positive cells. *B*, human OS cells (MG63, MNNG/HOS and SaOS2) were treated with ZOL at the indicated doses for 48h. *C*, mRNA extracts were analyzed by real-time PCR for CLU. Protein extracts were analyzed by western blotting for CLU and vinculin expression. All experiments were repeated at least three times. ***, p<0.001; ** p<0.01.

First we assessed the effects of ZOL treatment on CLU expression *in vivo* in HOS-MNNG osteosarcoma xenografts using immunohistochemistry (Fig. 1A). Once tumors became palpable, mice were treated with ZOL and tumors were harvested for immunohistochemical analysis. While ZOL rapidly and significantly induced CLU expression (18h after treatment; Supp. Fig.1), CLU expression increased 2.5-fold after treatment with ZOL (***, p<0.001) compared with vehicle treated tumor after 3 weeks of treatment (Fig. 1A), suggesting that ZOL treatment induced a stress response characterized by this increase of CLU expression. In addition, increased expression of CLU is durable 7 days after ZOL withdrawal (Supp. Fig. 1).

To confirm these *in vivo* results, dose-dependent effects of ZOL on the expression of CLU mRNA and protein levels were evaluated in a panel of human osteosarcoma cell lines (MNNG/HOS, MG63 and SaOS2). ZOL significantly increased CLU mRNA level in a dose-dependent manner depending of the tumor cell lines (Fig. 1B). CLU protein levels also increased in a dose-dependent manner after ZOL treatment in all tested osteosarcoma cell lines, as analyzed by western-blotting (Fig. 1C).

### ZOL-resistant cells overexpress CLU and CLU overexpression protects osteosarcoma tumor cells from ZOL inhibitory effect

**Figure 2:**
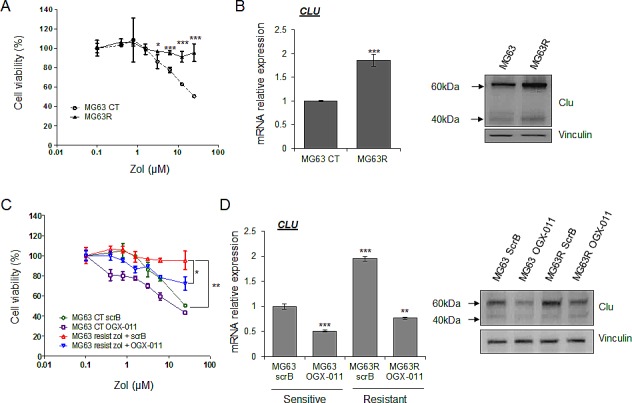
CLU induced by ZOL treatment protects tumor cells and leads to ZOL resistance MG63 cells were treated with increased doses of ZOL for 6 months to become resistant to ZOL (MG63R or MG63 resist.). After this time, the selected cells were ‘pooled’, in order to avoid clonality. *A*, MG63R and MG63 were treated with ZOL for the indicated doses for 48h. Cell growth was determined by crystal violet and compared with control. *B*, mRNA extracts (*left panel*) and protein extracts (*right panel*) of MG63 versus MG63R were analyzed by real-time PCR or by western blotting for CLU and vinculin expression. *C*, MG63 and MG63R cells were treated twice with 300nM OGX-011 or control ScrB ASO, followed by the indicated concentration of ZOL for 48h. Cell growth was determined by crystal violet and compared with control. *D*, MG63 and MG63R cells were treated twice with 300nM OGX-011 or control ScrB ASO. mRNA extracts (*left panel*) and protein extracts (*right panel*) of MG63 versus MG63R were analyzed by real-time PCR or by western blotting for CLU and vinculin expression. All experiments were repeated at least three times. *** p<0.001; ** p<0.01; * p<0.05.

As CLU is overexpressed after ZOL treatment and is known to confer resistance to treatment in various cancers, we hypothesized that CLU could be involved in the emergence and maintenance of resistance of osteosarcoma cells to ZOL treatment. Based on this hypothesis, MG63 and HOS-MNNG tumor cells were cultured in presence of increasing concentration of ZOL for 6 months to obtain ZOL-resistant cells, called MG63R and HOS-MNNG-R cells. After this time, the selected cells were ‘pooled’, in order to avoid clonality. We first compared the sensitivity of MG63 and HOS-MNNG sensitive cells versus MG63R and HOS-MNNG-R cells in presence of ZOL. While ZOL treatment significantly inhibited cell viability of MG63 sensitive cells in a dose-dependent manner, no significant effect was observed on MG63R (Fig. 2A, *right panel*), showing that these cells became completely resistant to ZOL at the tested doses. The same results were obtained with HOS-MNNG-R cells (Supp. Fig. 2A). Then, we observed that MG63R exhibited a higher CLU expression at both RNA and protein level than MG63 sensitive cells (Fig. 2B), suggesting an association between CLU levels and the resistance to ZOL in OS cells.

Then, we tried to re-sensitize resistant cells to ZOL treatment by knocking down CLU using OGX-011 antisense oligonucleotide (ASO), a specific CLU inhibitor (Fig. 2C and D). First we observed that MG63R cells (in red) treated with control ScrB are still resistant to ZOL treatment compared with MG63 sensitive cells treated with ScrB ASO (in green; Fig. 2C). However, CLU inhibition using OGX-011 significantly but partially re-sensitzed MG63R tumor cells (in blue) to ZOL inhibitory effect by decreasing cell viability by 25% compared with MG63R treated with ScrB ASO (Fig. 2C). Moreover, MG63 sensitive cells treated with OGX-011 (in violet) are more sensitive to ZOL treatment than the MG63 sensitive cells treated with ScrB ASO (in green), suggesting a benefit effect of combination of OGX-011 with ZOL by inhibiting CLU expression (Fig. 2C and D). These results were confirmed by clonogenic assay in MG63 and HOS-MNNG resistant cells (Supp. Fig. 2B). These results suggest that CLU is involved in the emergence of resistance to ZOL. Moreover, CLU knock-down prevents this emergence of resistance and enhance ZOL activity to inhibit OS cell growth.

Confirming the idea that CLU confers resistance to ZOL, we then transiently overexpressed CLU in osteosarcoma cells. CLU mRNA and protein levels were analyzed osteosarcoma cells (Supp. Fig. 2C). CLU mRNA expression was significantly increased to 4.5-fold compared to control cells and confirmed by western blot analysis (Supp. Fig. 2C). Then, the response to ZOL treatment was evaluated in these osteosarcoma CLU-overexpressed cells versus the control cells by a cell viability assay. While control cells responded to ZOL treatment in a dose-dependent manner, ZOL decreased also the cell viability in a dose-dependent manner in cells CLU-overexpressing cells (Supp. Fig. 2C). However, cells CLU-overexpressed cells were significantly less sensitive to ZOL treatment than the control cells, demonstrating that CLU overexpression protects osteosarcoma cells to ZOL-induced cytotoxicity (Supp. Fig. 2C).

### CLU regulates the resistance phenotype of osteosarcoma cells

**Figure 3:**
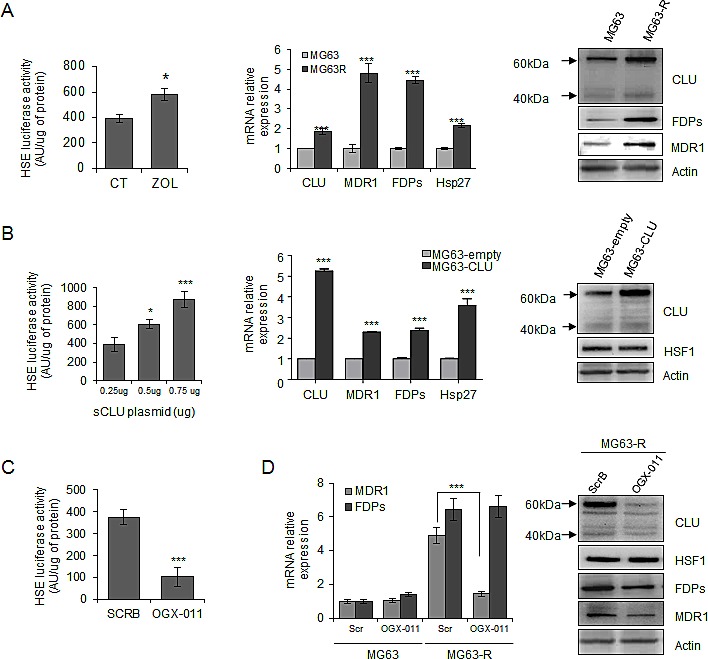
Clusterin protects tumor cells from ZOL-induced effects via a regulation of HSF1 activity and MDR1 expression *A*, *left panel*, MG63 cells were transiently transfected with HSE-luciferase plasmid, followed by 10μM ZOL treatment for 48h. Cells were harvested and HSE-luciferase activity was evaluated and represented in arbitrary units per μg of protein. *Middle and right panels*, mRNA and protein extracts of MG63 and MG63R cells were analyzed for CLU, MDR1, FDPs, Hsp27 and Actin expression by qPCR and western blotting, respectively. *B, left panel*, MG63 cells were transiently transfected with indicated concentrations of CLU-plasmid (Total amount of plasmid DNA transfected was normalized to 0.75μg per well by the addition of an empty vector), followed by transfection of HSE-luciferase plasmid for 48h. Cells were harvested and HSE-luciferase activity was evaluated and represented in arbitrary units per μg of protein. *Middle panel*, MG63 cells were transiently transfected to overexpress CLU (MG63-CLU) compared with empty vector (MG63-empty) and mRNA extracts were analyzed for CLU, MDR1, FDPs and Hsp27 expression. *Right panel,* Protein extracts were analyzed for CLU, HSF1 and Actin expression by western blotting. *C*, MG63 cells were transiently transfected with HSE-luciferase plasmid, followed by 300nM OGX-011 or ScrB ASO twice for 48h. Cells were harvested and HSE-luciferase activity was evaluated and represented in arbitrary units per μg of protein. *D,* MG63R and MG63 cells were treated twice with 300nM OGX-011 or ScrB for 48h. *Left panel*, mRNA extracts were analyzed for MDR1 and FDPs expression. *Right panel*, protein extracts were analyzed for CLU, HSF1, FDPs, MDR1 and Actin expression by western-blotting. All experiments were repeated at least three times. ***, p<0.001; *, p<0.05.

Because HSF1 is a master regulator of the heat shock response [30], we then evaluate the effect of ZOL treatment on HSF1 activity and HSPs expression. First, we used the Heat Shock Element (HSE) reporter designed to monitor the activity of heat shock response through measuring the transcriptional activity of HSF1, leading to induction of Hsps and CLU expression. We first verified that overexpression of HSF1 increased HSE luciferase activity (Supp. Fig. 3A). Moreover, it is well known that exposure of cells to elevated temperatures such as 42°C activates HSF1 binding to HSE, which induces HSF1-driven reporter activity (Supp. Fig 3B). Then, we found that ZOL significantly induced HSE luciferase activity suggesting that ZOL increased HSF1 transcriptional activity (Fig. 3A, *left panel*) and CLU expression (Fig. 1). In MG63R cells, enhanced CLU expression is accompanied with increased-expression level of Hsp27 compared with MG63 sensitive cells (Fig. 3A, *middle panel*), suggesting an increase of heat shock response induced by HSF1.

We know that CLU overexpression protects tumor cells from ZOL-induced cell death (Supp. Fig. 2C), and we found that overexpression of CLU also increased HSF1 activity in a dose dependant-manner (Fig. 3B, *left panel*) followed by increase of Hsp27 expression (Fig. 3B, *middle panel*), without affecting HSF1 expression (Fig. 3B, *right panel*). Moreover, CLU knockdown using OGX-011 significantly decreased HSF1 transcriptional activity (Fig. 3C) without affecting HSF1 expression (Fig. 3D, *right panel*), which was also confirmed by using specific siRNA targeting CLU (Supp. Fig. 3C), while knock-down of HSF1 using siRNA decreased CLU expression (Supp. Fig. 3C). All These data suggest a feed-forward regulation of HSF1 by CLU as we previously reported in prostate cancer [31].

Resistance to ZOL treatment involves different biological processes including increase expression of MDR1 and FDPs [32], as observed in MG63R tumor cells compared with MG63 sensitive cells (Fig. 3A, *middle and right panel*) and in HOS-MNNG-R compared with HOS-MNNG sensitive cells (Supp. Fig. 3D). Surprisingly, this increase of MDR1 and FDPs expressions correlates with the increase of CLU expression in MG63R cells. Moreover, overexpression of CLU in MG63 cells also leads to significant increase of MDR1 and FDPs expressions (Fig. 3B, *middle panel*), suggesting that CLU could regulate MDR1 and FDPs expressions. MDR1 is reported to be transcriptionally regulated directly by HSF1 with the presence of HSE sequence in MDR1 promoter [33], and we effectively confirm that HSF1 knockdown using siRNA significantly reduced MDR1 expression while FDPs expression was not affected (Supp. Fig. 3E). Interestingly, CLU inhibition using OGX-011 significantly reduced MDR1 expression in MG63R, but did not affect FDPs expression at transcriptional level compared with MG63R treated with ScrB ASO (Fig. 3D, *left panel*), while both FDPs and MDR1 expressions were decreased at protein level (Fig. 3D, *right panel*). All these results suggest that CLU indirectly regulates MDR1 expression at transcriptional level by regulating HSF1 activity (Fig. 3C). However, CLU inhibition does not affect FDPs expression at transcriptional level but seems to regulate FDPs at protein level probably by its chaperone activity.

### OGX-011 enhances ZOL induced apoptosis in osteosarcoma cell lines

**Figure 4:**
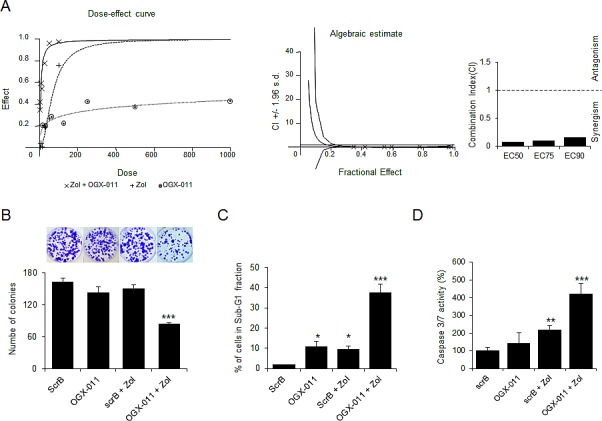
CLU knockdown enhances effects of ZOL treatment in OS cells *A*, Dose-dependent effects and Combination Index (CI) values calculated by CalcuSyn software were assessed in MNNG/HOS cells treated for 48 hours with OGX-011 alone, ZOL alone, or combined treatment at indicated concentration with constant ratio design between both drugs. Cell growth was determined by crystal violet. The CI for ED_50_, ED_75_ and ED_90_ was respectively 0.084, 0.104 and 0.162 indicating a synergistic effect (lower than 1) of CLU inhibition combined with ZOL. *B,* osteosarcoma cells were treated with 300nM OGX-011 or ScrB ASO +/− 1μM ZOL for 2 days, and then plated at clonal density for colony counts. *C*, HOS cells were treated with 300nM OGX-011 or ScrB ASO +/− 1μM ZOL for 2 days and the proportion of cells in subG_1_ was determined by propidium iodide staining. *D*, Cells were harvested, and Caspase-3/7 activity was determined on the cell lysates and the results are expressed in arbitrary units and corrected for protein content (*D*). All experiments were repeated at least three times. *** p<0.001; ** p<0.01; * p<0.05.

Since ZOL induces up-regulation of CLU and CLU-functions as mediator in treatment resistance [28, 29, 34], we next evaluated whether CLU knockdown potentiated the effect of ZOL treatment. To determine whether this effect was additive or synergistic, the dose-dependent effects with constant ratio design and the combination index (CI) values were performed and calculated according to the Chou and Talalay median effect principal [35]. Figure 4A shows the dose response curve (combination treatment, OGX-011 or ZOL monotherapy) and the combination index plots, indicating that OGX-011 synergistically enhances the effect of ZOL on HOS-MNNG tumor cell growth (Fig. 4A). These results were confirmed in MG63, SaOS2 and U2OS cells (Supp. Fig. 4).

Then we performed a colony formation assay to evaluate capabilities to recover after 2 days of OGX-011 +/-ZOL treatment. While neither OGX-011 nor ZOL did not change the number of colonies compared with control ScrB ASO (Fig. 4B), the combination of OGX-11 with ZOL significantly decreased the colony formation compared with each single drug alone (Fig. 4B).

Moreover, OGX-011 potentiates the effect of ZOL inhibitor to induce apoptosis (Fig. 4C and D). Flow cytometric analysis shows that apoptotic rates (subG1 fraction) significantly increased (p<0.001) when OGX-011 is combined with ZOL (38.4%) compared to control ScrB (1.8%), OGX-011 (10.8%), control ScrB ASO + ZOL (9.3%; Fig. 4C). The significant increase of caspase-3/7 activity confirms that OGX-011 sensitizes cells to ZOL inhibition with increased apoptotic rates (Fig. 4D).

### OGX-011 potentiates zoledronic acid activity in MNNG/HOS xenografts *in vivo*

**Figure 5:**
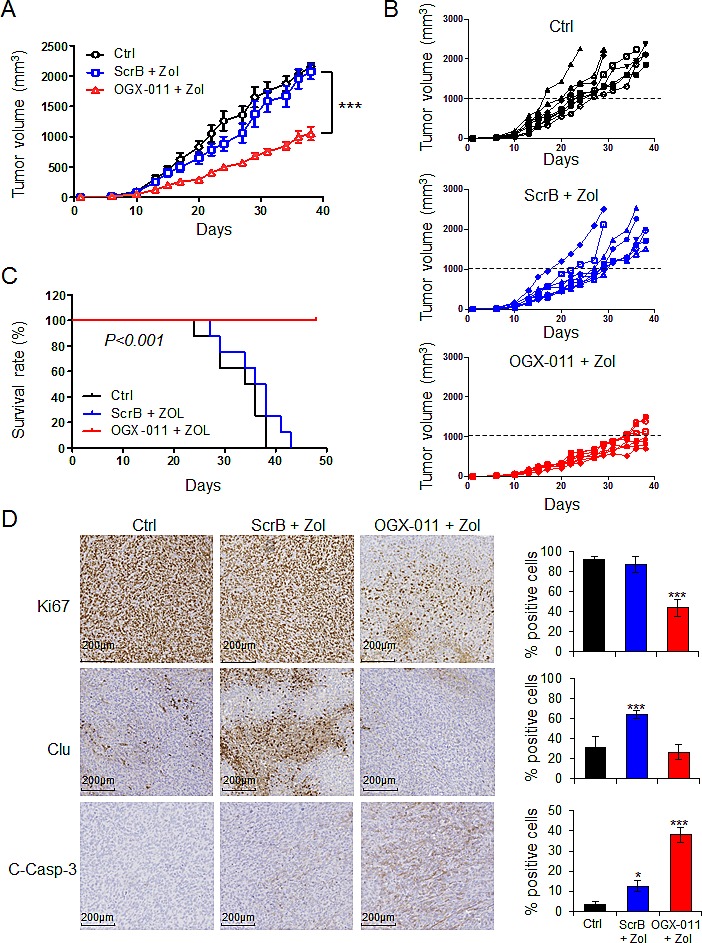
OGX-011 potentiates ZOL activity in MNNG/HOS osteosarcoma xenograft model Mice were treated twice a week with 50μg/kg ZOL and 15mg/kg OGX-011 starting when tumors were palpable as described in M&M. The mean tumor volume (*A*) and individual tumor volume (*B*) were compared between the 3 groups ± SEM (n=8). ***, p<0.001. *C*, in Kaplan-Meier curve, cancer-specific survival was compared between the 3 groups over a 46-d period. ***, p<0.001. *D*, tumors were collected and CLU, Ki67, and cleaved-caspase-3 were evaluated by immunohistochemical analysis. Specimens were scored and estimated in percentage of positive cells. The control group corresponds to the mice bearing tumor that did not receive any treatment. This group only received vehicle. * p<0.05; *** p<0.001.

The effects of combined treatment with OGX-011 and ZOL were evaluated in HOS-MNNG xenograft tumors. Athymic mice were injected with human osteosarcoma HOS-MNNG cells in paratibial site. Once tumors were palpable, mice were randomly assigned to vehicle control, ZOL + ScrB ASO, or ZOL + OGX-011 groups. In a first *in vivo* experiment, we confirmed that ScrB and OGX-011 do not have effect on tumor growth compared with tumor control group (Supp. Fig. 5A and B). In a second experiment, all animals treated with ZOL + OGX-011 (n=8) had significant delays in tumor growth compared with other groups starting at day 15 (respectively 196 mm^3^ for ZOL + OGX-011 versus 451.3 mm^3^ for control and 404.9 mm^3^ for ZOL + ScrB ASO) and after 36 days (respectively 997.2 mm^3^ for ZOL + OGX-011 versus 2023.6 mm^3^ for control and 1954.2 mm^3^ for ZOL + ScrB ASO; ***, p<0.001; Fig. 5A). Until day 27, ZOL + ScrB shows marginal but not significant decrease of tumor growth compared with control group (respectively 1368.5 mm^3^ and 1649.9 mm^3^ at day 27). Moreover, at individual level, 0 out of 8 mice developed tumor with volume over 1000mm^3^ at day 30, while all mice in control and ZOL + ScrB ASO groups exhibited tumor volume over 1000mm^3^ (Fig. 5B). Overall survival was significantly prolonged in mice treated with combined ZOL + OGX-011 (p<0.001; Fig. 5C). By day 42, all mice died or were euthanized due to high tumor burden in control and in ZOL + ScrB groups as compared with the combined ZOL + OGX-011 group, where all mice were still alive after 46 days. These data demonstrate that targeting CLU using OGX-011 potentiates the effects of ZOL to significantly inhibit tumor growth and prolongs survival in osteosarcoma xenograft model.

Consistent with *in vitro* findings, immunohistochemical analysis reveals decreased ZOL-induced CLU, and Ki67 expression after treatment with combined ZOL + OGX-011 compared with other groups (Fig. 5D), corroborating the *in vitro* results. Ki67 expression correlates with the mean tumor volume (Fig. 5A and D). Indeed, ZOL + OGX-011 treatment delayed tumor growth and decreased Ki67 expression compared with other groups exhibiting both high tumor volume and elevated Ki67 expression. Additionally, tumors treated with ZOL + OGX-011 combination had significant higher apoptosis rates compared with other groups as shown by increased cleaved-Caspase-3 staining (Fig. 5D). These data suggest that decreases in tumor progression in ZOL + OGX-011 treated tumors result from both reduced proliferation rates as well as increased apoptosis rates.

### CLU knock-down does not ameliorate ZOL-induced bone prevention

**Figure 6:**
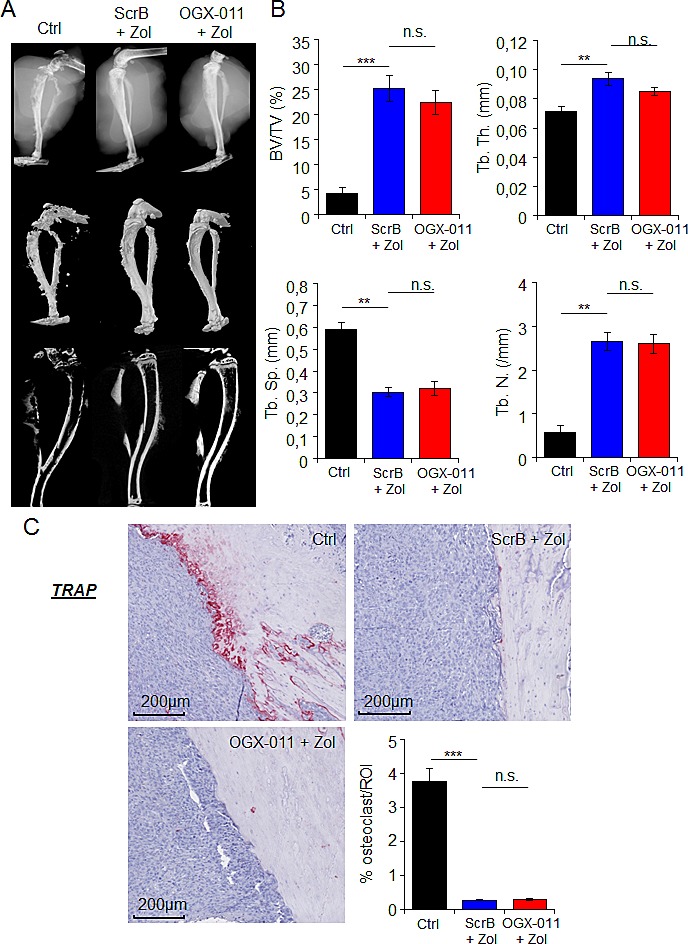
Effect of combination treatment on bone prevention in MNNG/HOS osteosarcoma xenograft model *A*, representative radiographic, microCT, and 3D model images of the tumor-bearing tibia taken *ex vivo*, from HOS-MNNG xenograft model treated with OGX-011 (15mg/kg) + ZOL (50μg/kg), ScrB ASO + ZOL or control (Ctrl; ScrB + PBS). *B*, MicroCT quantification of the specific trabecular bone volume (BV/TV (%)) (*upper left panel*), trabecular thickness (Tb.Th (mm)) (*upper right panel*), trabecular separation (Tb.Sp (mm)) (*lower left panel*) and trabecular number (Tb.N (/mm)) (*lower right panel*) were calculated on the tibia of tumor-bearing mice of the different groups. *C*, TRAP expression was evaluated by immunohistochemical analysis. Specimens were scored and the surface occupied (in %) by osteoclast was determined by ImageJ in the delimited ROI. *** p<0.001; ** p<0.01; n.s., not significant.

ZOL is known to prevent bone lesions and indirectly inhibit tumor growth by blocking the vicious cycle between bone resorption and tumor cells within the tumor-bone microenvironment [36]. That is why we first evaluate the effect of combined treatment on bone lesions on tumor-bearing tibia in HOS-MNNG xenograft model. The bone micro architecture parameters of the tumor-bearing tibia, from MNNG/HOS xenograft model treated with ZOL + SCRB, ZOL + OGX-011 or vehicle have been measured using X-ray, microCT and 3D reconstruction models (Fig. 6A). Data analysis revealed a decrease with ZOL treatment of the tumor-associated osteolysis that we usually observe on the tumor-bearing bone (Fig. 6A and B). An extensive analysis of multiple bone parameters revealed a strong and significant increase of tumor-associated bone quality in the ZOL treated groups (ZOL + SCRB, ZOL + OGX-011) compared to control group. Indeed, ZOL treatment increased significantly the trabecular bone volume relatively to the tissue volume (BV/TV) from 4.2% to 25.2% and 22.5% respectively in ZOL + SCRB and ZOL + OGX-011 groups, the trabecular number (Tb.N) from 0.56 to 2.64 and 2.6mm, the trabecular thickness (Tb.Th) from 0.07 to 0.093 and 0.085mm and decreased the trabecular separation (Tb.Sp) from 0.59 to 0.3 and 0.31mm (Fig. 6B).

Histological analysis of the tumor-bearing bone with TRAP staining revealed a significant decreased of surface occupied by osteoclasts compared with control group in presence of ZOL (ZOL + SCRB and ZOL + OGX-011 groups; Fig. 6C). However, no significant difference was observed between ZOL + SCRB and ZOL + OGX-011 groups. OGX-011 does not improve ZOL-induced bone prevention of tumor-bearing tibia compared to ZOL alone; suggesting that the significant decrease of tumor growth is due to a direct inhibition of tumor cells and not via inhibition of bone prevention. Consequently, OGX-011 seems to potentiate the direct effect of ZOL to inhibit tumor cells in HOS-MNNG xenograft model.

## DISCUSSION

Cancer cells express high levels of molecular chaperones and pirate the protective functions of HSF1 to support their transformation [37]. Molecular chaperones help cells cope with stress-induced protein aggregation, and play prominent roles in cell signaling and transcriptional regulatory networks. A growing enthusiasm for therapeutic modulation of this proteostasis network highlights Hsp's and CLU as rational targets because of their multifunctional roles in signaling and transcriptional networks associated with cancer progression and treatment resistance. In this study, we set out to evaluate the role of CLU in this heat shock response in OS since CLU is dramatically induced by ZOL treatment and CLU inhibitor is currently in phase III clinical Trial in prostate cancer. We report that ZOL induces a stress response with activation of the transcription factor HSF1 and subsequent increased levels of CLU. This heat shock response likely enhances emergence of treatment resistance, as Hsp27 inhibition using siRNA which attenuates ZOL-mediated Hsp27 expression and potentiates the effect of ZOL *in vitro* [20].

CLU expression is rapidly up-regulated in various cancer tissues, including osteosarcoma [23]. Previous studies have also linked CLU expression with induction and progression of many cancers [29]. Consistent with these accumulated findings [38], inhibition of CLU using OGX-011 synergistically enhances conventional as well as molecular targeted therapies in cancer preclinical models [31, 39]. Here, we show that ZOL increases CLU levels by enhancing HSF1 transcriptional activity both *in vitro* and *in vivo*, while OGX-011 inhibits ZOL induced CLU expression. We also found that CLU silencing abrogates, while CLU overexpression enhances, ZOL-induced HSF1 transcriptional activity, suggesting a role for CLU in the regulation of HSF1 and the heat shock response itself, as reported in the literature [31]. This effect of CLU on HSF1 activity is biologically relevant since CLU overexpression protects, while CLU silencing enhances, ZOL-induced cytotoxicity in OS cells. Consistent with our *in vitro* results, synergistic effects were also observed *in vivo* in HOS-MNNG xenograft models when OGX-011 was combined with ZOL. OGX-011 and ZOL combination significantly delays OS tumor growth and prolonged survival in HOS-MNNG xenograft model.

ZOL is known to prevent bone lesions and indirectly inhibit tumor growth by blocking the vicious cycle between bone resorption and tumor cells within the tumor-bone microenvironment [36]. Indeed, by blocking osteoclasts, ZOL inhibits the release of growth factor (TGF-β, IGF-1…) trapped in bone matrix and liberated during bone resorption to activate proliferation of tumor cells. In our experiment, OGX-011 does not improve ZOL-induced bone prevention of tibia bearing-tumor in HOS-MNNG xenograft model compared to ZOL alone; suggesting that the significant decrease of tumor growth after combined treatment is due to a direct inhibition of tumor cells and not via inhibition of bone prevention. Consequently, OGX-011 strongly potentiates the direct effect of ZOL to inhibit tumor cells in OS xenograft model. Indeed, decreased Ki67 expression and increased apoptotic rates with combined ZOL and CLU inhibition suggests that delayed tumor progression resulted from both inhibition of tumor proliferation and enhanced treatment-induced apoptosis. These *in vivo* data are consistent with the *in vitro* data, and are the proof of principle to combine OGX-011 with ZOL as new therapeutic strategy in treatment of OS.

Molecular mechanisms of acquired drug resistance often involve expression of one or more energy-dependent transporters that detect and eject anticancer drugs from cells [17]. Studies on mechanisms of cancer drug resistance have yielded important information about how to circumvent this resistance to improve cancer treatment. So far, few studies have reported molecular mechanisms underlying N-BPs-induced resistance. Kars *et al.* reported that increased ABC transporters expression (BCRP and LRP), as well as up-regulation of the anti-apoptotic Bcl-2 gene were found in ZOL-resistant breast cancer cells as compared to sensitive cells [40]. In the current study, we show for the first time an increase of another ABC transporter, MDR1 in ZOL-resistant MG63 and HOS-MNNG cells compared with sensitive cells. MDR1 promoter has been shown to contain heat shock elements (HSE) sequence [33], and to be transcriptionally regulated by HSF1 [41]. Consequently, inhibition of heat shock factor response using quercetin decreased MDR1 expression [41]. We found that ZOL induced HSF1 activity thus increasing MDR1 expression, while HSF1 knockdown using siRNA reduced MDR1 expression. While CLU is known to be transcriptionally activated by HSF1 [29, 31], in this study we also show that CLU exerts a feed forward loop that in turn activates HSF1, and also maintain MDR1 expression. Indeed, transient overexpression of CLU significantly increased MDR1 expression. On the other hand, CLU knockdown using OGX-011 decreases HSF1 transcriptional activity, which subsequently leads to decreased MDR1 expression, similar to that observed after HSF1 knockdown. Collectively, these results highlight a biologically relevant feed-forward regulation loop of CLU on HSF1 activity and, indirectly on MDR1 regulation (Supp. Fig. 6).

Ory *et al.* described one other molecular mechanism of ZOL resistance in OS cells by indicating that prolonged treatment with ZOL increased FDPs expression [32], a critical enzyme involved in the mevalonate pathway that is inhibited by N-BPs, while Milone *et al.* contested this hypothesis [42]. In our study, we confirmed that ZOL-resistant cells exhibited higher FDPs expression than the sensitive cells and, this result was correlated with increase CLU expression. However, we demonstrated that CLU does not affect FDPs expression at transcriptional level, but decreases FDPs expression at protein level suggesting that the molecular chaperone CLU could regulate FDPs pathway at protein level, and this need further investigations.

Moreover, several studies have reported a potential role of p38-MAPK pathway and/or its downstream target Hsp27 in the resistance of N-BPs treatment in preclinical models of breast cancer and osteosarcoma [10, 20, 43]. In our study we found that ZOL treatment induced HSF1 activity thus enhancing at least, Hsp27 expression according with the literature. However, we also demonstrated that CLU, also induced by HSF1, regulates via the feed-forward loop, HSF1 activity and thus, indirectly regulates Hsp27 expression that could lead to other molecular mechanisms inducing resistance.

The variety of described molecular mechanisms underlying N-BPs-induced resistance could be attributed to the heterogeneity of tumor cells or the methods used for inducing resistance. We described some molecular mechanisms of resistance regulated by CLU, but it is not excluded that other more complex mechanisms take part in the emergence of ZOL resistance. Thus, the main problem is to determine which molecular mechanism is involved for each patient, in order to adapt the best therapy in the era of personalized medicine.

In addition to synergistically enhancing anti-tumor activity, combination therapy of OGX-011 + ZOL may also allow dose reduction strategies to reduce toxicity and undesirable side effects such as osteonecrosis of jaw that has been associated with ZOL in clinical trials and clinical use. In the present study, 50μg/kg ZOL monotherapy, corresponding to the dose used in young patients with OS in accord with the literature [44], showed marginal, non-significant decreases in tumor volume but a significant effect on bone prevention; however, significant delays in tumor progression were seen at this dose when ZOL was combined with OGX-011, with no toxicity observed. Moreover, OGX-011 is a second-generation ASO with a long tissue half-life of ~7 days that suppresses CLU levels *in vitro* and *in vivo*. OGX-011 improved the efficacy of many varied anti-cancer therapies by suppressing treatment-induced CLU and the stress response [31] and increased pre-clinical activity in many xenograft models of cancer [29, 39, 45].

In summary, this paper helps define how stress induced by ZOL regulates CLU by induction of HSF1 activity and, in turn, how CLU regulates HSF1 activity, cell survival, and treatment resistance. We demonstrate, for the first time, that CLU inhibition abrogates the heat shock response and synergistically potentiates the cytotoxic activity of ZOL directly on tumor cells, thus, preventing emergence of resistance. These observations are clinically relevant since CLU inhibitors are in phase III clinical trials in prostate cancer, and provide a framework for building new drug combinations built on mechanism-based interventions to overcome drug resistance. The present study supports for the first time the development of targeted strategies employing OGX-011 in combination with ZOL to improve patient outcome in osteosarcoma.

## MATERIALS AND METHODS

### Tumor cell lines

The human osteosarcoma cell lines MG63 (young male osteosarcoma), SaOS2 (young female osteosarcoma), U2OS (young female osteosarcoma from tibia origin) and MNNG/HOS (young female high grade osteosarcoma from femur origin transformed in vitro by N-methyl N'-nitro-N-nitrosoguanidine treatment), were purchased from the American Type Culture Collection and maintained in DMEM (Invitrogen-Life Technologies, Inc.) supplemented with 5% fetal bovine serum and 2mmol/L L-glutamine. All cell lines were cultured in a humidified 5% CO_2_/air atmosphere at 37^o^C. All cell lines were passaged for less than 3 months after resurrection.

### Therapeutic agents

ZOL, 1-hydroxy-2-(1*H*-imidazole-1-yl) ethylidene-bisphosphonic acid supplied as the disodium salt by Novartis Pharma AG, was dissolved in PBS at 10mM stock solution and stored at −20°C.

Second-generation antisense (OGX-011) and scrambled (ScrB) oligonucleotides with a 2′-*O*-(2-methoxy) ethyl modification were supplied by OncoGenexPharmaceuticals (Vancouver, British Columbia, Canada). OGX-011 sequence 5′-CAGCAGCAGAGTCTTCATCAT-3′ corresponds to the initiation site in exon II of human CLU. The ScrB control sequence was 5′-CAGCGCTGACAACAGTTTCAT-3′. siRNA targeting HSF1 or CLU and siRNA control (siSCR) were purchased from SantaCruz Biotechnology (Dallas, USA). Osteosarcoma cells were treated with siRNA or oligonucleotides using protocols described previously [39].

### Cell proliferation and apoptosis assays

Osteosarcoma cancer cell lines were plated in DMEM with 5% FBS and treated with zoledronic acid at indicated concentration and time point, cell growth being measured using the crystal violet assay as described previously [46]. Detection and quantitation of apoptotic cells were done by Caspase-3 assay (described below). Each assay was repeated in triplicate.

The combination index (CI) was evaluated using CalcuSyn dose effect analysis software (Biosoft, Cambridge, UK). This method, based on the multiple drug effect equation of Chou-Talalay [35], is suitable for calculating combined drug activity over a wide range of growth inhibition: CI =1, additivity; CI >1, antagonism; CI <1, synergism. CI was calculated at ED_50_ and ED_75_.

Caspase-3/7 activity was assessed 3 days after treatment using the kit CaspACE Assay System, Fluorometric (Promega, Madison, WI, USA). Fifty μg of total cell lysate were incubated with caspase-3 substrate AC-DEVD-AMC at room temperature for 4h and caspase-3 activity was quantified in a fluorometer with 360nm excitation and 460nm emission.

### Cell cycle analysis

Osteosarcoma cell lines were treated twice with 300nM ScrB or OGX-011, followed by 1μM ZOL for 48h, trypsinized, washed twice and incubated in PBS containing 0.12% Triton X-100, 0.12mM EDTA and 100μg/ml ribonuclease A; 50μg/ml propidium iodide was then added to each sample for 20min at 4°C. Cell cycle distribution was analyzed by flow cytometry (Beckman Coulter Cytomics FC-500, Beckman, Inc., Miamai, FL), based on 2N and 4N DNA content. Each assay was done in triplicate.

### Western blotting analysis

Samples containing equal amounts of protein (depending on the antibody, 5-50μg) from lysates of cultured tumor OS cell lines underwent electrophoresis on SDS-polyacrylamide gel and were transferred to nitrocellulose filters. The filters were blocked in PBS containing 3% BSA and 0.1% Tween at room temperature for 1h and blots were probed overnight at 4°C with primary antibodies (anti-clusterin, Santa Cruz Biotechnology, Dallas, TX, USA; anti-vinculin, Sigma-Aldrich Corp, St. Louis, MO, USA) to detect proteins of interest. After incubation, the filters were washed 3 times with washing buffer (PBS containing 0.1% Tween) for 5min. Filters were then probed with the secondary antibody coupled to horseradish peroxidase. Antibody binding was visualized with the enhanced chemiluminescence system (Roche Molecular Biomedicals).

### Quantitative Reverse Transcription-PCR

Total RNA was extracted from cultured cells (after 48h of treatment) using TRIzol reagent (Invitrogen Life Technologies, Inc.). Two μg of total RNA was reversed transcribed using the Transcriptor First Strand cDNA Synthesis Kit (Roche Applied Science). Real-time monitoring of PCR amplification of complementary DNA (cDNA) was performed using DNA primers (supplemental table S1) on ABI PRISM 7900 HT Sequence Detection System (applied Biosystems) with SYBR PCR Master Mix (Applied Biosystems). Target gene expression was normalized to GAPDH levels in respective samples as an internal standard, and the comparative cycle threshold (Ct) method was used to calculated relative quantification of target mRNAs. Each assay was performed in triplicate.

### Luciferase assay

Osteosarcoma cells (2.5×10^5^) were plated on six-plates and transfected using lipofectin (6μL per well; Invitrogen Life Technologies, Inc.). The Heat Shock Element (HSE) reporter is designed to monitor the activity of heat shock response through measuring the transcriptional activity of HSF1. The total amount HSE plasmids DNA used were normalized to 1μg per well by the addition of a control plasmid. Ten μM zoledronic acid was added 8h after the transfection and for 48h. HSE-luciferase activity was measured using Dual-Luciferase Reporter Assay System (Promega) with the aid of a microplate luminometer (TriStar LB-941 Berthold Technologies). All experiments were carried out in triplicate wells and repeated 3 times.

### Animal Treatment

Five-week-old female Rj:NMRI-nude mice were anesthetized by inhalation of an isoflurane/air mixture (2%, 1L/min) before an i.m. inoculation of 2 × 10^6^ human MNNG/HOS osteosarcoma cells close to the tibia. Tumors appeared in contact with the tibia approximately 8 days later and led to osteolytic lesions associated with development of pulmonary metastasis mimicking the human pathology. Once tumors were palpable, mice were randomly assigned (n=8) to vehicle, ZOL + ScrB ASO or ZOL + OGX-011 groups. ZOL (50μg/kg; formulation in PBS) was injected s.c. three times per week and OGX-011 or ScrB ASO (15mg/kg) was injected intra-peritoneally once daily for the first week and then three times per week. Each experimental group consisted of 8 mice. Tumor volume was measured three times weekly (length × width × depth × 0.5432). Data points were expressed as average tumor volume ± SEM.

When tumor volume reached ≥10% of body weight, mice were sacrificed and tumors harvested for immunohistochemistry. Animal care and experimental protocols were approved by the French Ministry of Research and were done in accordance with the institutional guidelines of the French Ethical Committee protocol agreement number 1280.01 and under the supervision of authorized investigators.

### Immunohistochemistry

Immunohistochemical stains were performed on formalin-fixed and paraffin-embedded 3μm sections of tumor samples using adequate primary antibody, and the Ventana autostainer Discover XT (Ventana Medical System) with enzyme labeled biotin streptavidin system and solvent resistant 3,3′-diaminobenyidine Map kit. All comparisons of staining intensities were made at 200× magnifications.

### Statistical analysis

All *in vitro* data were assessed using the Student t test and Mann-Whitney test. Tumor volumes of mice were compared using Kruskal-Wallis test. Overall survival was analyzed using Kaplan-Meier curves and statistical significance between the groups was assessed with the log-rank test (Graphpad Prism). Levels of statistical significance were set at *P*<0.05.

## SUPPLEMENTARY MATERIAL FIGURES



## Abbreviations

OS: osteosarcoma
HSP: heat shock proteins
CLU: clusterin
HSF1: heat shock factor 1
ZOL: zoledronic acid
ASO: antisense oligonucleotide
MDR1: multidrug-resistant 1
FDPs: farnesyl diphosphate synthase
