# Supplementary material for: Clusterin inhibition using OGX-011 synergistically enhances zoledronic acid activity in osteosarcoma
Source: Oncotarget. 2014 Aug 4;5(17):7805–19. doi: 10.18632/oncotarget.2308 (PMC4202162; doi:10.18632/oncotarget.2308)
Supplement: Supplementary file 1 [file oncotarget-05-7805-s001.pdf]

Clusterin inhibition using OGX-011 synergistically enhances zoledronic acid activity in osteosarcoma

Supplementary Material

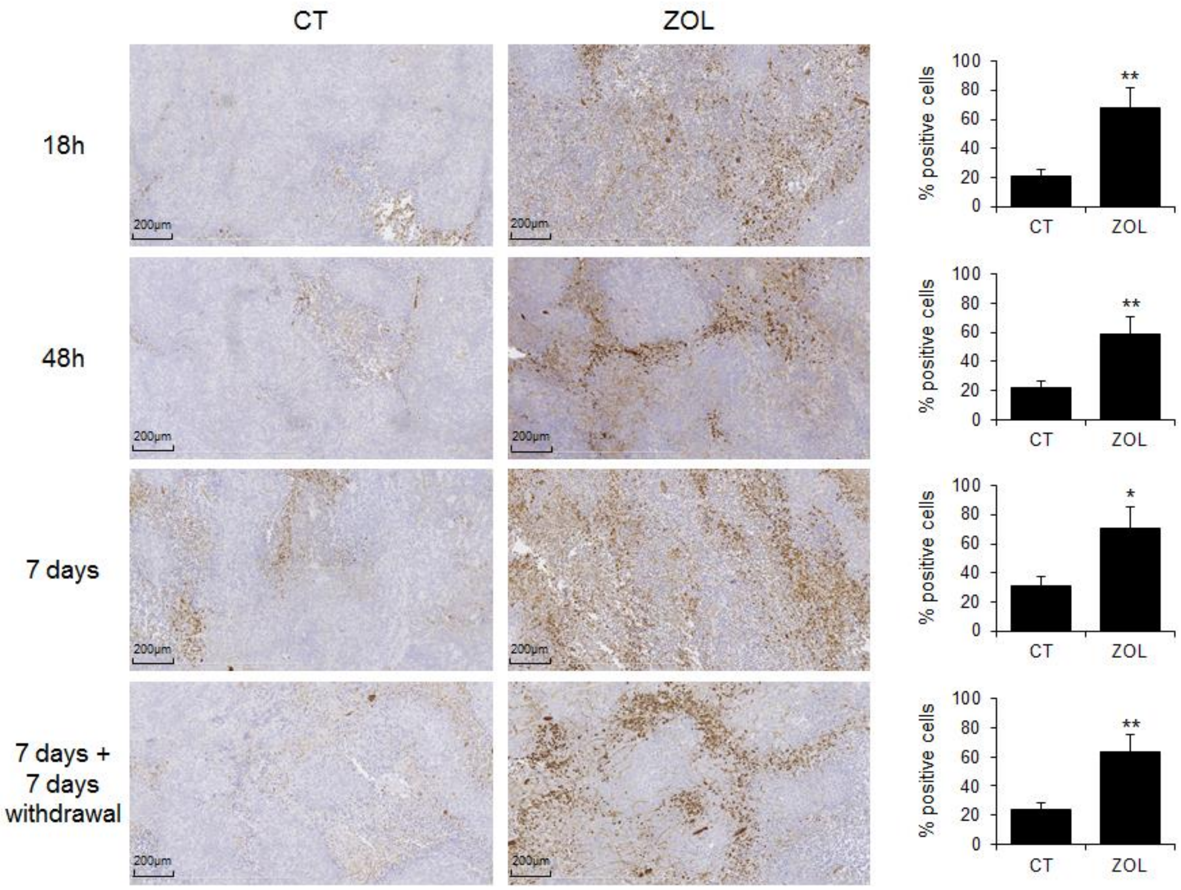

Supp. Figure 1: ZOL induces CLU expression in osteosarcoma xenograft model. HOS-MNNG tumor cells were injected in paratibial site. Once tumors were palpable, mice were randomly assigned to vehicle (PBS) or ZOL (50ug/kg, s.c.) treated every two days. Mice were treated for 18h, 48h, 7 days or 7 days followed by withdrawal of ZOL for 7 days. Tumors were collected and CLU expression was evaluated by immunohistochemical analysis. Specimens were scored and estimated in % positive cells. ** $p<0.01$; * $p<0.05$.

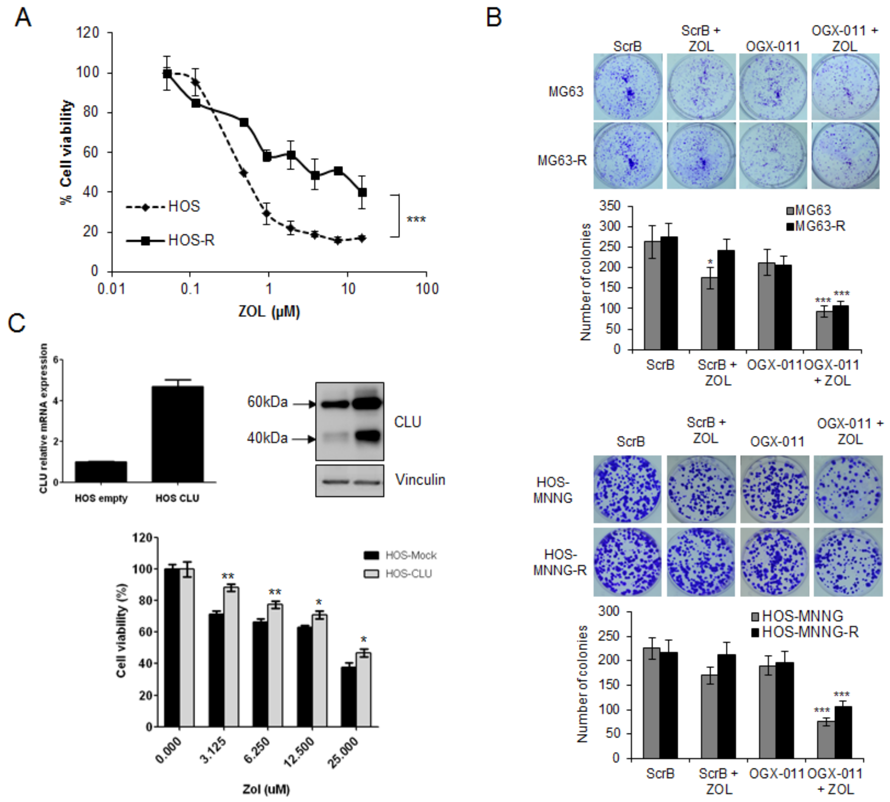

Supp. Figure 2: **CLU inhibition sensitizes resistant tumor cells to ZOL, while transient overexpression of CLU protects osteosarcoma tumor cells from ZOL inhibitory effect.** *A*, HOS-MNNG cells were treated with increased doses of ZOL for 6 months to become resistant to ZOL (HOS-MNNG-R). After this time, the selected cells were 'pooled', in order to avoid clonality. HOS-MNNG-R and HOS-MNNG were treated with ZOL for the indicated doses for 48h. Cell growth was determined by crystal violet and compared with control. *B*, MG63/MG63R and HOS-MNNG/HOS-MNNG-R cells were treated twice with 300nM OGX-011 or control ScrB ASO, followed by 10µM ZOL for 48h. Then, the cells were plated without any treatment at clonal density for colony counts after coloration with crystal violet. *C*, MNNG/HOS osteosarcoma cells were transiently transfected with empty- or CLU-plasmid for 48h to overexpress CLU, versus an empty vector as a control (-mock). mRNA extracts were analyzed by real-time PCR for CLU expression. Protein extracts were analyzed by western blotting for CLU and vinculin expression. Tumor cells were treated with ZOL for the indicated doses for 48h. Cell growth was determined by crystal violet and compared with control. All experiments were repeated at least three times. *** $p<0.001$; ** $p<0.01$, * $p<0.05$.

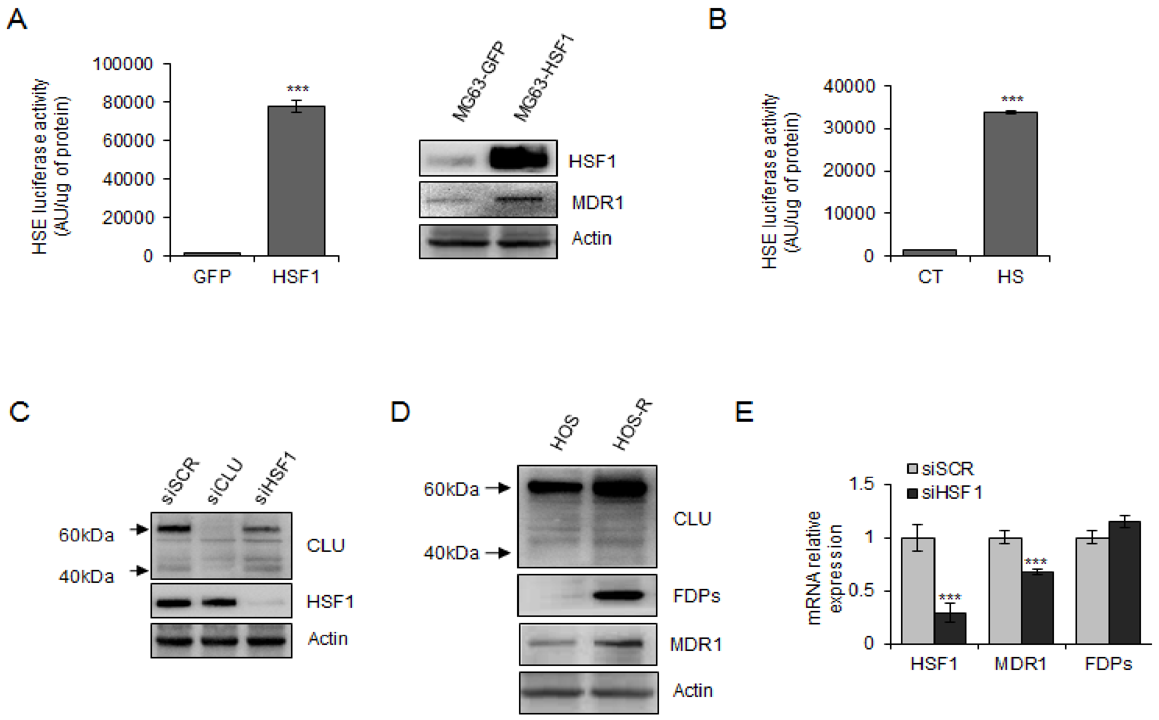

Supp. Figure 3: HSF1 activity and MDR1 expression. *A*, *Left panel* MG63 cells were transiently co-transfected with HSF1-plasmid or GFP vector as control and with HSE-luciferase plasmid for 48h. Cells were harvested and HSE-luciferase activity was evaluated and represented in arbitrary units per µg of protein. *Right panel*, protein extracts were analyzed for HSF1, MDR1 and Actin expression by western blotting. *B*, MG63 cells were transiently transfected with HSE-Luciferase plasmid and after 48hours, the cells were incubated at 42°C for 45min (Heat Shock: HS) followed by 1h recovery time at 37°C. Then, the cells were harvested and HSE-luciferase activity was evaluated and represented in arbitrary units per µg of protein. *C*, tumor cells were treated with 30nM HSF1 siRNA, or CLU siRNA vs control Scr siRNA for 48h. Protein extracts were analyzed for HSF1, CLU and Actin expression by western blotting. *D*, protein extracts of HOS-MNNG and HOS-MNNG-R cells were analyzed for CLU, MDR1, FDPs and Actin expression by western blotting *E*, MG63R cells were treated with 30nM HSF1 siRNA vs control Scr siRNA for 48h. mRNA extracts were analyzed for HSF1, MDR1 and FDPs expression. *** $p<0.001$.

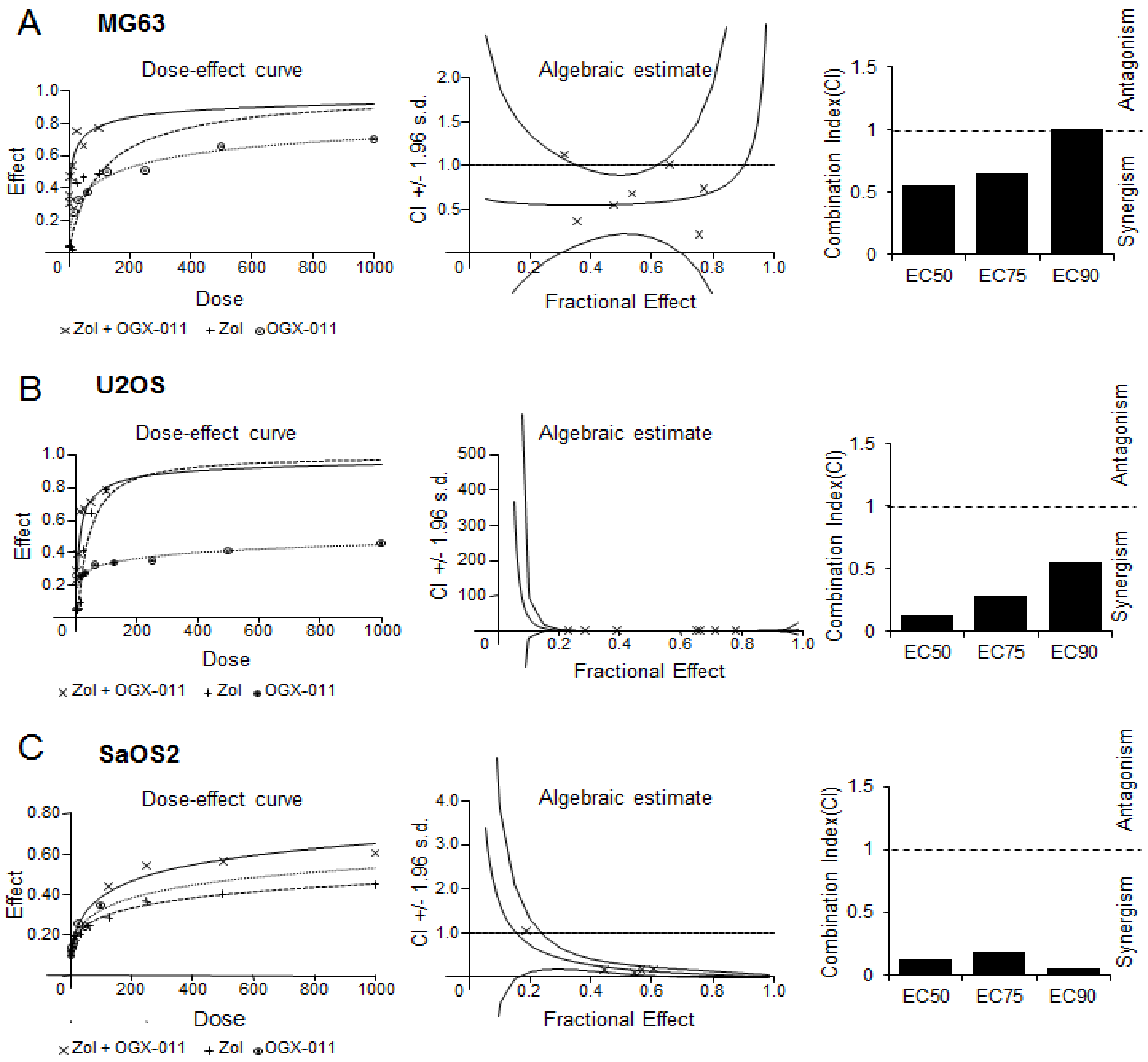

Supp. Figure 4: CLU knockdown enhances effects of ZOL treatment in OS cells. *A*, Dose-dependent effects and Combination Index (CI) values calculated by CalcuSyn software were assessed in MG63 (*A*), U2OS (*B*) and SaOS2 (*C*) cells treated for 48 hours with OGX-011 alone, ZOL alone, or combined treatment at indicated concentration with constant ratio design between both drugs. Cell growth was determined by crystal violet. The CI for ED_{50}, ED_{75} and ED_{90} were significantly lower than 1, indicating a synergistic effect of CLU inhibition combined with ZOL. All experiments were repeated at least thrice.

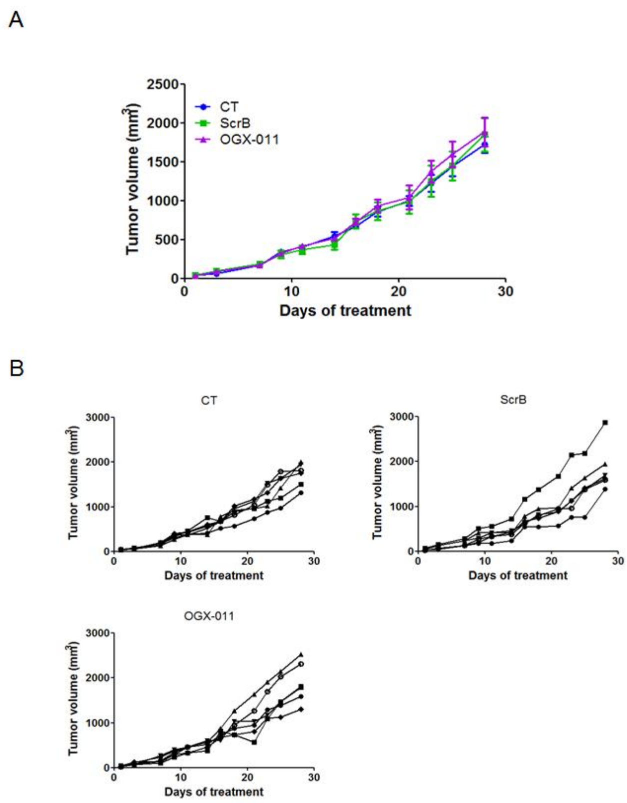

Supp. Figure 5: Effect of OGX-011 in MNNG/HOS osteosarcoma xenograft model. Mice were treated with 15mg/kg OGX-011 or ScrB ASO starting when tumors were palpable as described in M&M. The mean tumor volume (*A*) and individual tumor volume (*B*) were compared between the 3 groups ± SEM (n=6).

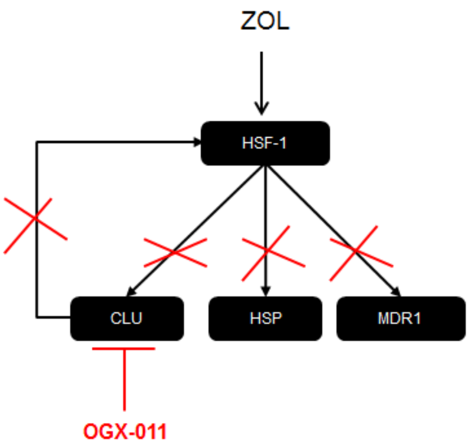

Supp. Figure 6: The feed-forward regulation loop of CLU on HSF1 activity and, indirectly on MDR1 regulation.
